# Phase–amplitude coupling and the BOLD signal: A simultaneous intracranial EEG (icEEG) - fMRI study in humans performing a finger-tapping task

**DOI:** 10.1016/j.neuroimage.2016.08.036

**Published:** 2017-02-01

**Authors:** T. Murta, U.J. Chaudhary, Tim M. Tierney, A. Dias, M. Leite, D.W. Carmichael, P. Figueiredo, L. Lemieux

**Affiliations:** a Dept. of Clinical and Experimental Epilepsy, UCL Institute of Neurology, London, United Kingdom; b Institute for Systems and Robotics and Department of Bioengineering, Instituto Superior Técnico, Universidade de Lisboa, Lisboa, Portugal; c UCL Institute of Child Health, London, United Kingdom

**Keywords:** Intracranial EEG, fMRI, Phase–amplitude coupling, Electro-haemodynamic coupling

## Abstract

Although it has been consistently found that local blood-oxygen-level-dependent (BOLD) changes are better modelled by a combination of the power of multiple EEG frequency bands rather than by the power of a unique band alone, the local electro-haemodynamic coupling function is not yet fully characterised. Electrophysiological studies have revealed that the strength of the coupling between the phase of low- and the amplitude of high- frequency EEG activities (phase–amplitude coupling - PAC) has an important role in brain function in general, and in preparation and execution of movement in particular. Using electrocorticographic (ECoG) and functional magnetic resonance imaging (fMRI) data recorded simultaneously in humans performing a finger-tapping task, we investigated the single-trial relationship between the amplitude of the BOLD signal and the strength of PAC and the power of α, β, and γ bands, at a local level. In line with previous studies, we found a positive correlation for the γ band, and negative correlations for the PAC_βγ_ strength, and the α and β bands. More importantly, we found that the PAC_βγ_ strength explained variance of the amplitude of the BOLD signal that was not explained by a combination of the α, β, and γ band powers. Our main finding sheds further light on the distinct nature of PAC as a functionally relevant mechanism and suggests that the sensitivity of EEG-informed fMRI studies may increase by including the PAC strength in the BOLD signal model, in addition to the power of the low- and high- frequency EEG bands.

## Highlights

• First study of single-trial correlations between the phase amplitude coupling strength and BOLD.
• Intracranial EEG and fMRI data simultaneously recorded in humans during a motor task.
• PAC_βγ_ strength explains variance of BOLD in addition a combination of α, β, and γ band powers.

## 1 Introduction

In the last century, human brain activity has been recorded most commonly as electrical potentials on the scalp (scalp electroencephalography - EEG), neocortex (electrocorticography - ECoG), or inside the brain (depth EEG). Since the early 1990s, recording changes in the local blood oxygenation, using blood-oxygen-level-dependent (BOLD) functional magnetic resonance imaging (fMRI), has become an increasingly important tool, largely due to its non-invasive whole-brain coverage and relatively high spatial resolution. However, notwithstanding numerous studies on the electrophysiological correlates of the BOLD effect, our understanding of the coupling between EEG and BOLD signals remains limited (Valdes-Sosa et al., 2009, Murta et al., 2015).

Since the discovery of EEG, a range of rhythmic activities characteristically associated with sensory, motor, and cognitive events, has been observed on its recordings (Engel et al., 2001, Varela et al., 2001, Jacobs and Kahana, 2010). These activities appear to hierarchically interact with each other, as the “basic units” of a complex system that regulates information processing in the brain, across multiple spatial and temporal scales (Lakatos et al., 2005, Palva et al., 2005, Roopun et al., 2008, Canolty and Knight, 2010, Buzsaki et al., 2012, Hyafil et al., 2015). The influence of the phase of the low-frequency (LF) activity in the amplitude of the high-frequency (HF) activity, a phenomenon called phase–amplitude coupling (PAC_LF HF_), has attracted great interest due to its potential functional role (Kramer et al., 2008, Lakatos et al., 2008, Cohen et al., 2009a, Cohen et al., 2009b, Tort et al., 2009, Axmacher et al., 2010, Buzsáki et al., 2012). In the human motor cortex, EEG activity above 40 Hz is increased during movement, whereas α (8–14 Hz) and β (14–30 Hz) activities tend to be decrease during its execution, in comparison to rest (Crone et al., 1998, Miller et al., 2012). Fluctuations in PAC_αγ_ and PAC_βγ_ have also been linked to movement execution and pre- and/or post- movement rest (Miller et al., 2012, Yanagisawa et al., 2012).

With regard to fMRI, it has been found that EEG power fluctuations in the α, β, and γ bands explain independent, as well as common, components of the BOLD signal amplitude variance (Scheeringa et al., 2011, Magri et al., 2012). Many studies have also investigated the relationship between multi-unit activity (MUA), local field potentials (LFP) and the BOLD signal. While some found that both MUA and LFP were equally good correlates of the BOLD signal amplitude (Mukamel et al., 2005, Nir et al., 2007), others found that LFP accounted for significantly larger amounts of its variance (Logothetis and Wandell, 2004). It is currently accepted that the BOLD signal reflects both LFP and MUA, to different degrees, depending on the conditions (Ekstrom, 2010). Of particular relevance to us is the observation by Whittingstall and Logothetis (2009) who found that the γ band power was a good predictor of MUA only when its increases were time-locked to a certain δ phase, suggesting that a particular interaction between the phase and the amplitude of different bands may predict MUA better, and it may therefore also be related to the BOLD signal differently.

In summary, (i) fluctuations in α, β, and γ band power, and in PAC_αγ_ and PAC_βγ_ strength, are reflections of the brain state; (ii) fluctuations in the power of multiple frequency bands predict BOLD changes better than fluctuations in the power of the γ band alone; (iii) LFP and MUA predict BOLD changes differently, depending on the conditions; and (iv) PAC fluctuations predict MUA better than LFP γ power fluctuations alone.

In this study, we used ECoG and fMRI data simultaneously recorded in humans performing a finger-tapping task to investigated how single-trial fluctuations in the PAC strength relate to the co-localised single-trial fluctuations in the amplitude of the BOLD signal; in particular, to investigate whether fluctuations in the PAC strength explained variance of the BOLD signal amplitude that was not explained by a combination of α, β, and γ band powers. Using invasive EEG recordings (ECoG, depth EEG) is a way to surpass one of the major limitations of scalp EEG - the presence of the skull between the recording site and the active neuronal tissue that acts as a low-pass filter of the electrical field potential, which therefore allowed to record EEG activity above 70 Hz reliably. It is also a way to guarantee that we are investigating the relationship between the electrophysiological and haemodynamic responses at a local level (the responses under investigation are most likely to be generated by the same brain region). Furthermore, using invasive EEG and fMRI data simultaneously recorded is the only way to guarantee that we are analysing responses related to the same (neuronal) phenomenon. Single-trial variability due to different performances, habituation effects, plasticity, or uncontrolled variations in the response to the stimulation paradigm cannot be investigated using data sequentially acquired (Villringer et al., 2010). More importantly, the patient’s determination and physical capability to perform the task exactly when asked to do it do not affect dramatically studies using data simultaneously acquired but they do affect studies using sequentially acquired data, which potentially results in a lower correlation between the two responses that is not explained by their decoupling. We have recently used this type of data to study the BOLD correlates of sharp waves; we found that the amplitude of the BOLD signal depends more on the duration of the underlying field potential (reflected in the sharp wave width) than on the degree of neuronal activity synchrony (reflected in the sharp wave amplitude) (Murta et al, 2016). To the best of our knowledge, this is the first study investigating single-trial correlations between the strength of PAC, estimated from human ECoG data, and the amplitude of the BOLD signal simultaneously recorded, at a local level.

## 2 Methods

### 2.1 Patient selection

Intracranial EEG (icEEG) and fMRI data were simultaneously recorded in patients (of either sex) with severe drug-resistant epilepsy, who underwent invasive EEG monitoring as part of their pre-surgical evaluation. The implantation scheme and electrophysiological (depth EEG and/or ECoG) data acquired varied across patients, depending on their clinical history and surgical considerations. In total, seven patients had part of or the whole motor cortex covered by subdural strips and/or grids. The icEEG-fMRI data were acquired with the written informed consent of patients, as part a project approved by the Joint UCL/UCLH Committees on the Ethics of Human 107 Research, at the neuroradiology department of the National Hospital for Neurology and Neurosurgery in London, following strict guidelines (Carmichael et al., 2008, Carmichael et al., 2010).

### 2.2 Simultaneous acquisition of icEEG and fMRI data

The icEEG-fMRI acquisition consisted of two 10 min resting state runs and a 5 min motor task run. In this study, we focus entirely on the latter. Six patients alternatively tapped their left- and right- fingers, in blocks of 30 s each. In two cases, the finger-tapping blocks were interleaved with 30 s blocks of rest. The seventh patient performed the same task, with rest, using his feet. The simultaneous icEEG-fMRI data acquisition protocol is described in Carmichael et al. (2012).

The MRI data was acquired on a 1.5 T scanner (TIM Avanto, Siemens, Erlangen, Germany), with a quadrature head transmit–receive radio-frequency (RF) coil using low specific absorption rate sequences (<0.1 W/kg head average), simultaneously with icEEG data, in accordance with our acquisition protocol (Carmichael et al. 2012).

The fMRI scan consisted of a gradient-echo echo-planar imaging (GE-EPI) sequence with the following parameters: TR/TE/flip angle=3000 ms/40ms/90°, 64×64 acquisition matrix, 38×2.5 mm slices with 0.5 mm gap. In addition, a FLASH T1 weighted structural scan was acquired with the following parameters: TR/TE/flip angle=15 ms/4.49 ms/25°, resolution 1.0×1.2×1.2 mm, FoV 260×211×170 mm, 256×176×142 image acquisition matrix with the readout direction lying in the sagittal plane; scan duration: 6 min 15 s.

Details on safety concerns of recording icEEG data simultaneously with fMRI data and on fMRI data quality were previously discussed in Carmichael et al. (2010) and Carmichael et al. (2012), respectively.

IcEEG data were acquired with an MR-compatible system (Brain Products, Gilching, Germany) and respective software (Brain Recorder, Brain Products, Gilching, Germany), at a 5 kHz sampling rate. The icEEG recording system was synchronised with the 20 kHz gradient MR scanner clock.

The computed tomography (CT) data was acquired with a Siemens, SOMATOM Definition AS+ scanner, with a 0.43×0.43×1mm resolution and a 512×512×169 image matrix, shortly after the implantation of the icEEG electrodes and prior to the icEEG-fMRI acquisition, as part of the patients' clinical management.

### 2.3 Pre-processing of icEEG and fMRI data

SPM12 (http://www.fil.ion.ucl.ac.uk/spm/software/spm12/) was used to realign and spatially smooth (using an isotropic 5 mm FWHM Gaussian kernel) the fMRI data. Prior to smoothing, physiological noise was removed from the fMRI data using FIACH (Functional Image Artefact Correction Heuristic) (Tierney et al., 2015). In brief, FIACH is a two-step biophysically-based approach designed to identify brain regions that show high temporal instability and to remove large amplitude temporal BOLD signal changes of non-neuronal origin. Based on the fMRI signal from the brain regions identified in the first step, FIACH generates 6 regressors that reflect physiological noise and can be included in the GLM as confounds.

After removing the MR acquisition-related artefacts from the icEEG data using an average template subtraction approach (Allen et al., 2000), the icEEG data were down-sampling to 500 Hz. No hearbeat-related artefact correction was necessary as previously reported (Carmichael et al., 2012).

In this study, we use the specific terms ECoG or depth EEG rather than the generic term icEEG to refer to the electrophysiological signal when suitable.

Bipolar icEEG time courses were obtained for every pair of adjacent icEEG contacts by subtraction of the voltage of the more posterior contact from that of the more anterior one.

### 2.4 Functional MRI motor function mapping and the BOLD time-course of interest

A square wave function corresponding to the periods of contralateral (to the icEEG contacts) finger tapping was convolved with the canonical HRF and used as regressor of interest in a whole-brain general linear model (GLM) analysis of the pre-processed fMRI data. The following confounding effects were also included in this model, as regressors of no interest: 24 movement related confounds (6 realignment parameters, and their Volterra expansion (Friston et al., 1996)), and 6 fMRI physiological noise related confounds (Tierney et al., 2015). A square-wave function representing periods of ipsilateral (to the icEEG contacts) finger tapping was convolved with the canonical HRF and also included in the model, in the cases where finger tapping was interleaved with rest. All GLM were estimated using SPM12 (http://www.fil.ion.ucl.ac.uk/spm/software/spm12/).

A positive t-contrast for the regressor of interest was used to localise the BOLD changes positively correlated with the contralateral (to the icEEG contacts) finger tapping. The corresponding statistical parametric maps were thresholded at p<0.001, uncorrected, and the resulting cluster with a minimum extent of 10 voxels, located in the motor cortex, was used as the region of interest (ROI) for the subsequent analyses. The BOLD signal time-course of interest was obtained by averaging the time-courses across the ROI voxels, and high-pass filtering the resulting time-course using a Butterworth filter of order 4 and cut-off frequency of 1/128 Hz (Matlab function *butter*), to remove the slow drift.

### 2.5 Contacts, frequency bands, and features of interest for BOLD modelling

This section describes various steps taken in the generation of the ECoG-derived features of interest, which were subsequently compared in terms of their capability to predict the co-localised finger-tapping related BOLD changes, i.e. the BOLD time course of interest.

We used the classical α (8–14 Hz), β (14–30 Hz), and γ (70–182 Hz) bands (Lopes da Silva, 2011) to identify the ECoG contact pairs that showed the largest task-related α, β, and γ power fluctuations (Miller et al., 2012); these were the contact pairs of interest - COI_α_, COI_β_, COI_γ_, and their search is further described in 2.5.1 Nevertheless, in the following steps of this study, we used patient-specific α and β bands, narrower than the classical bands, centred at patient-specific frequencies (Aru et al., 2014), and mainly containing rhythmic activity; their central frequencies were found as described in 2.5.2. Then, for each patient showing both significant finger tapping BOLD changes and a significant PAC effect, one or two (depending on whether the PAC effect was significant) PAC strength regressors - PAC_αγ_ and/or PAC_βγ_ - were computed, as described in 2.5.3.

#### 2.5.1 Contacts of interest (COI)

For each patient and ECoG contact pair located over the motor cortex (see Table 1 for a schematic illustration of these contacts), we computed the power spectra (using the Matlab function *fft*) over the two finger tapping periods: ipsilateral (to the icEEG contacts) finger tapping, $S_{\mathrm{ipsi}}\left(f\right)$, and contralateral finger tapping, $S_{\mathrm{contr}}\left(f\right)$. Then, for each frequency band of interest, fb=[f1,f2], we computed the difference between the areas under the two power spectra, ${∆S}_{\mathrm{fb}}$, as:

(2-1) $${∆S}_{\mathrm{fb}}=\left(\sum_{f=f1}^{f2}S_{\mathrm{contr}}\left(f\right)-\sum_{f=f1}^{f2}S_{\mathrm{ipsi}}\left(f\right)\right)∆f$$

where ∆f is the sampling frequency. The patient-specific COI were defined as those showing the largest ${∆S}_{\alpha }$, ${∆S}_{\beta }$, and ${∆S}_{\gamma }$, and were labelled COI_α_, COI_β_, and COI_γ_, respectively. Here, α and β were defined as the classical [8–14] Hz and [14–30] Hz frequency bands (Lopes da Silva, 2011), and γ as [70–182] Hz.

#### 2.5.2 Patient-specific frequency bands

Following the identification of the patient-specific COI_α_, COI_β_, and COI_γ_, we identified the central frequencies of the task-related and patient-specific α and β rhythmic activities in the harmonic component of the ECoG power spectrum. For each patient, we performed a coarse-graining spectral analysis (CGSA) (Yamamoto and Hughson, 1991) to isolate the fractal and harmonic components of the COI_α_ and COI_β_ power spectra (He et al., 2010); CGSA was applied to the blocks of ipsilateral (to the ECoG implantation) finger tapping to take advantage of the stronger α and β rhythmic activities during these periods.

First, the ECoG time course was segmented into 5 s non-overlapping epochs, which were multiplied by a Hanning window of the same length (obtained with the Matlab function *hann*), demeaned, and called x(i). Second, x(t), $x_{2}\left(t\right)$, and $x_{1/2}\left(t\right)$, were computed as:

(2-2) x(t)=x(i)(i=1,2,3,…,N/2)

(2-3) $$x_{2}(t)=x\left(i\right)(i=2,4,6,\ldots ,N)$$

(2-4) $$x_{1/2}\left(t\right)=x\left(i\right)(i=1,1,2,2,3,3,\ldots ,N/4)$$

where N is the number of data samples within each 5 s epoch. $x_{2}\left(t\right)$, and $x_{1/2}\left(t\right)$ are called the coarse-grained time courses. Third, the auto-power spectrum of x(t), $S_{\mathrm{xx}}$, the cross-power spectrum of x(t) and $x_{2}\left(t\right)$, $S_{xx_{2}}$, and the cross-power spectrum of x(t) and $x_{1/2}\left(t\right)$, $S_{xx_{1/2}}$, were obtained using Matlab functions *xcorr* and *fft*. Finally, the raw, the fractal, and the harmonic power spectra were computed as:

(2-5) $$S(f)\mathrm{raw}=\frac{1}{M}\sum_{m=1}^{M}S_{\mathrm{xx}}(f)$$

(2-6) $$S(f)\mathrm{fractal}=\sqrt{\frac{1}{M}\sum_{m=1}^{M}S_{xx_{2}}(f)∙\frac{1}{M}\sum_{m=1}^{M}S_{xx_{1/2}}(f)}$$

(2-7) S(f)harmonic=S(f)raw−S(f)fractal

where M is the number of epochs.

For each patient, the centre of the α band was chosen to be the frequency showing the maximum power in the band [8–14] Hz of the COI_α_ harmonic power spectrum, and its width to be 2 Hz; and the centre of the β band was chosen to be the frequency showing the maximum power in the band [14–30] Hz of the COI_β_ harmonic power spectrum, and its width to be 6 Hz (Aru et al., 2014). The γ band was kept as [70–182] Hz because no obvious peak was found in this frequency band of the COI_γ_ harmonic power spectrum.

#### 2.5.3 ECoG-derived BOLD predictors

In this section, we describe how the ECoG time courses were processed for PAC calculation, and how two PAC strength regressors, PAC_αγ_ and PAC_βγ_, were built for each patient.

##### 2.5.3.1 Band-pass filtering and Hilbert transform

As a necessary step for the computation of all the ECoG-derived features investigated in this study, the ECoG signals were band-pass filtered and Hilbert transformed.

The ECoG time courses were band-pass filtered using a 2-way-least squares Finite Impulse Response filter (EEGlab toolbox (http://sccn.ucsd.edu/eeglab/) function *eegfilt*), chosen because it limits phase distortion to a minimum (the input data was processed in both the forward and reverse directions). For the α and β ranges, the central frequencies of the filters were 8, 9, 10, …, 30 Hz (sampled at every 1 Hz), and their bandwidths were set to 1 Hz, to ensure a precise estimation of the instantaneous phase (Berman et al., 2012, Aru et al., 2014). For the γ range, the central frequencies were 70, 74, 78 …, 182 Hz (sampled at every 4 Hz), and their bandwidths were set to 60 Hz, twice the fastest β component, i.e. 30 Hz, to preserve the modulation that the instantaneous phase of β could have in the amplitude of γ (Berman et al., 2012, Aru et al., 2014).

Each band-passed ECoG time course was then transformed in the complex signal $x\left(t\right)=A(t)e^{i\varphi (t)}$, using the Hilbert transform (Matlab function *Hilbert*), where A(t) is the amplitude and φ(t) is the instantaneous phase of x(t).

##### 2.5.3.2 Phase–amplitude coupling (PAC) strength

The PAC strength was computed as proposed by Canolty et al. (2006), using the Matlab code made available online (Canolty et al. (2006) supplementary material). Let us define a composite signal $x_{\mathrm{composite}}\left(t\right)$ that combines the phase of a particular low-frequency component (LF), $\varphi_{\mathrm{LF}}\left(t\right)$, with the amplitude of a particular high-frequency, $A_{\mathrm{HF}}\left(t\right)$, such that:

(2-8) $$x_{\mathrm{composite}}\left(t\right)=A_{\mathrm{HF}}\left(t\right)e^{i\varphi_{\mathrm{LF}}\left(t\right)}$$

The raw strength of the coupling between $A_{\mathrm{HF}}\left(t\right)$ and $\varphi_{\mathrm{LF}}\left(t\right)$ in a particular epoch comprising T data samples was computed as:

(2-9) $$M_{\mathrm{raw}}=1/T\sum_{t=1}^{T}x_{\mathrm{composite}}\left(t\right)=1/T\sum_{t=1}^{T}A_{\mathrm{HF}}(t)e^{i\varphi_{\mathrm{LF}}(t)}$$

The z-scored strength of the coupling between $A_{\mathrm{HF}}\left(t\right)$ and $\varphi_{\mathrm{LF}}\left(t\right)$ was computed as the difference between $M_{\mathrm{raw}}$ and the mean of a distribution of surrogates of $x_{\mathrm{composite}}$, obtained by jittering $A_{\mathrm{HF}}\left(t\right)$ and $\varphi_{\mathrm{LF}}\left(t\right)$ by 200 random time lags, divided by the standard deviation of the same distribution of surrogates (Canolty et al., 2006). The mean and standard deviation of the distribution of surrogates were obtained using the Matlab function *normfit*. The z-scored PAC strengths were the subject of the subsequent analyses.

##### 2.5.3.3 PAC- and band power- based BOLD predictors

Two PAC strength- and three power- based regressors were built for BOLD modelling: PAC_αγ_, based on the α phase - γ amplitude coupling computed at COI_α_; PAC_βγ_, based on the β phase - γ amplitude coupling computed at COI_β_; P_α_, based on the α band power computed at COI_α_; P_β_, based on the β band power computed at COI_β_; P_γ_, based on the γ band power computed at COI_γ_.

**Fig. 1:**
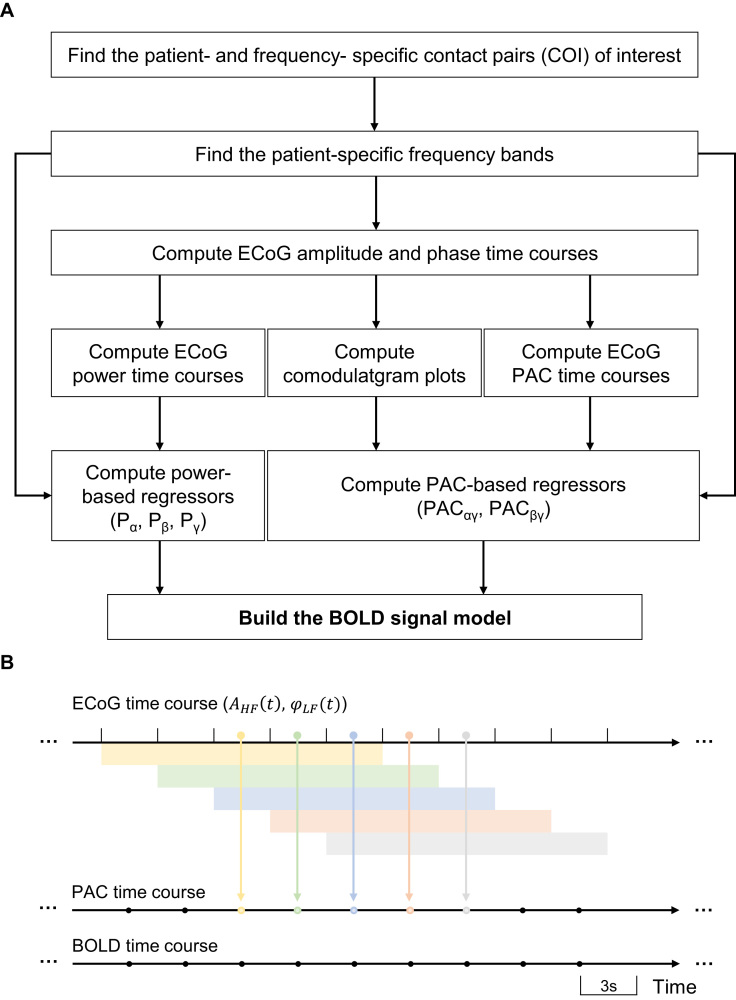
Schematic illustration of the processing steps used to build the BOLD signal model based on the ECoG signal. **A** PAC and power regressors computation pipeline. **B** Relationship between ECoG, PAC and BOLD time courses.

The computation of each PAC regressor involved: (i) the segmentation of COI_α_ and COI_β_ amplitude and phase complete time courses (Section 2.5.3.1) into 15 s overlapping epochs (5 TR), centred at the simultaneous BOLD signal epoch, (ii) the computation of a PAC strength estimate for each epoch; and (iii) the concatenation of the resulting PAC strength estimates, which resulted in a PAC strength regressor with the temporal resolution of the finger-tapping BOLD time course, i.e., 3 s (Fig. 1**B**). Tort et al. (2010) argued that 200 cycles of the low frequency of interest (that giving the phase) were enough to provide a reliable PAC strength estimate (in their particular experimental settings). We choose to use 15 s epochs, i.e., 120 cycles for the lower α component of interest (8 Hz), and 450 cycles for the higher β one (30 Hz). This seemed a good compromise between the accuracy of the PAC strength estimate (likely to increase with the number of cycles averaged) and the temporal smoothing (a consequence of the overlap).

The PAC strength metric proposed by Canolty et al. (2006) can be used to compute the strength of the coupling between a frequency pair, formed by the LF ECoG component giving the phase and the HF ECoG component giving the amplitude. After applying it to multiple frequency pairs, in parallel, the resulting z-scored PAC strengths (*z*-axis) can be plotted as a function of the LF giving the phase (*x*-axis) and the HF giving the amplitude (*y*-axis), in a form that it is often called the “phase–amplitude comodulogram” plot. In this study, these plots were used to improve the SNR of the PAC estimates. For this, they were computed using the patient specific- COI_α_ and COI_β_ complete ECoG time courses (5 min). Then, the PAC strength time courses whose frequency pairs showed a significant PAC (*p*<0.05; Bonferroni corrected for the dimensions of the frequency space) were averaged, which resulted in two average PAC strength time courses per patient. Finally, these two PAC strength time courses were convolved with the canonical HRF, which resulted in the two PAC strength regressors of interest, PAC_αγ_ and PAC_βγ_, respectively.

The power regressors of interest, *P*_α_, *P*_β_, and *P*_γ_, were obtained by: (i) averaging the power time courses over the corresponding patient-specific frequency bands and COI; (ii) convolving the average power time courses with the canonical HRF, and (iii) down-sampling the result to a 3 s (=TR) time resolution by averaging the power within each 3 s epoch.

### 2.6 BOLD model definition

We used the GLM framework to estimate the variance of the amplitude of the co-localised finger-tapping related BOLD changes, explained by each individual ECoG-derived effect (PAC, P_α_, P_β_, and P_γ_), in turn, which was not explained by a combination of the other regressors (e.g.: PAC vs [ P_α_ P_β_ P_γ_ ]).

**Fig. 2:**
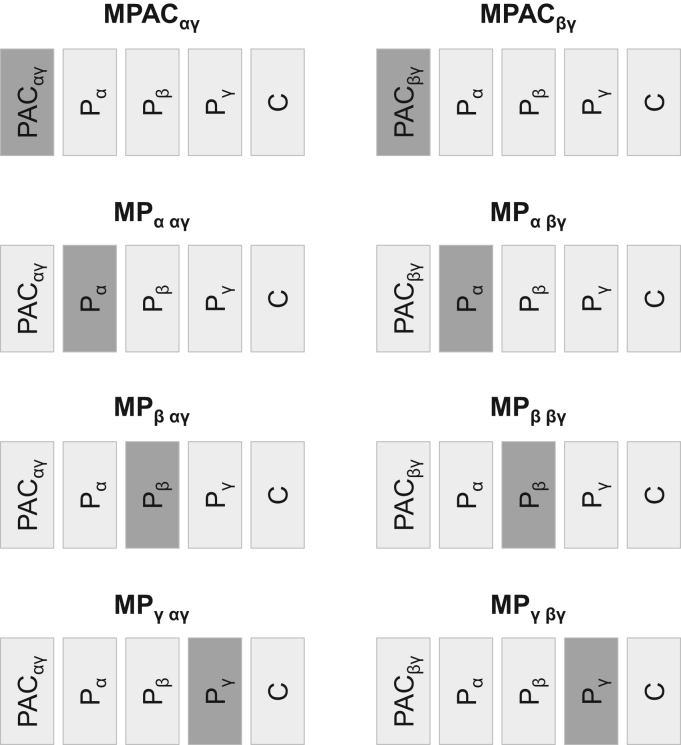
Design matrices of all the BOLD signal models considered. The regressor of interest, i.e. the regressor that was orthogonalised with respect to a combination of the others, is highlighted in dark grey. The “vertical banding artefacts” in the anatomical scans left hemispheres result from a processing error/setting that was made during the acquisition - each slice was scaled independently, which results in such stripy appearance.

We aimed to investigate the variance of the amplitude of the BOLD signal explained by the strength of PAC_αγ_ and PAC_βγ_ individually in addition to the standard model, i.e., that comprising the α, β, and γ power derived BOLD predictors. Eight independent models were built, four for each PAC-based regressor of interest (PAC_αγ_ and PAC_βγ_). Each model included a PAC-based regressor (PAC_αγ_ or PAC_βγ_); 3 ECoG power-based regressors (P_α_, P_β_, and P_γ_); and 30 confounding effects (C) (24 movement-related confounds (6 realignment parameters and their Volterra expansion (Friston et al., 1996)), and 6 fMRI physiological noise related confounds (Tierney et al., 2015). For example, the four linear models used to investigate the *PAC*_*αγ*_ effect were defined as follows:

(3-10) $$y=\beta_{{\mathrm{PAC}}_{\alpha \gamma }}\times \left({\mathrm{PAC}}_{\alpha \gamma }⊗\mathrm{HRF}\right)+\beta_{P_{\alpha }}\times \left(P_{\alpha }⊗\mathrm{HRF}\right){+\beta }_{P_{\beta }}\times \left(P_{\beta }⊗\mathrm{HRF}\right){+\beta }_{P_{\gamma }}\times \left(P_{\gamma }⊗\mathrm{HRF}\right)+\beta_{c}C+\epsilon$$

where ×, represents the product, ⊗, the convolution operation, y, the time course of the amplitude of the finger tapping BOLD signal (obtained as described in 2.4), HRF, the canonical HRF, C, the confounding effects matrix, ϵ, the error, β, the linear coefficients estimated for each regressor included in the model (${\mathrm{PAC}}_{\alpha \gamma }$, $P_{\alpha }$, $P_{\beta }$, $P_{\gamma }$, C), ${\mathrm{PAC}}_{\alpha \gamma }$, the time course of the strength of PAC_αγ_, $P_{\alpha }$, the time course of the power of γ, etc_…_ The models were labelled: MPAC_αγ_, MP_α,αγ_ , MP_β,αγ_, MP_γ,αγ_ and MPAC_βγ_, MP_α,βγ_, MP_β,βγ_, MP_γ,βγ,_ and their design matrices are illustrated in Fig. 2, where the regressor of interest, i.e. the regressor that was orthogonalised with respect to the combination of the others, is highlighted in dark grey. This orthogonalisation was necessary to assure that we were estimating the amount of variance that was explained by a particular regressor but not by a set of others. Note that (1) all eight models have the same exact number of regressors, and (2) each set of four models (one set for PAC_αγ_, other for PAC_βγ_) explains the same variance of the BOLD signal amplitude (the only difference being the way this explained variance is distributed among the regressors).

All models were estimated using the Matlab function *glmfit*.

## 3 Results

### 3.1 Functional MRI motor function mapping and the BOLD time-course of interest

**Fig. 3:**
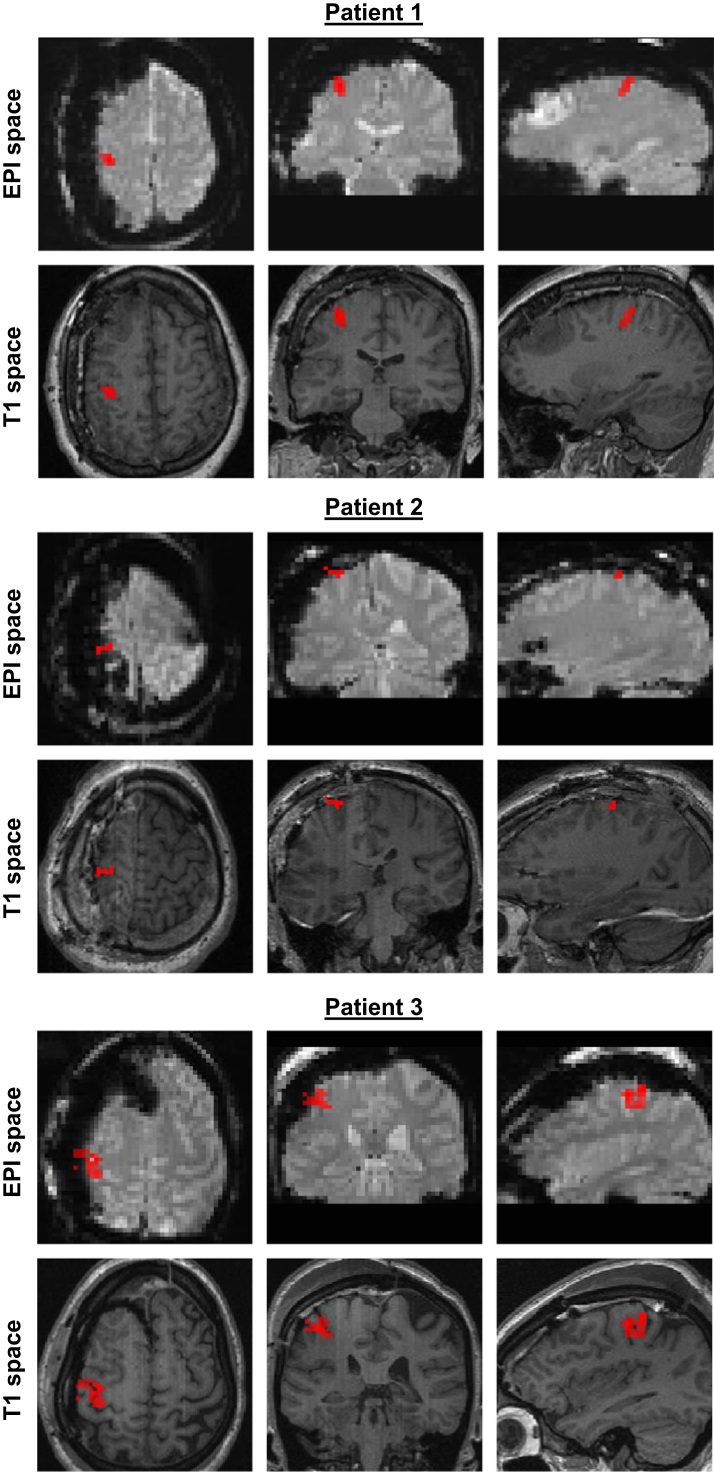
BOLD changes of interest. Statistical parametric maps of positive finger-tapping related BOLD changes (t-contrast), thresholded at *p*<0.001, uncorrected.

**Fig. 4:**
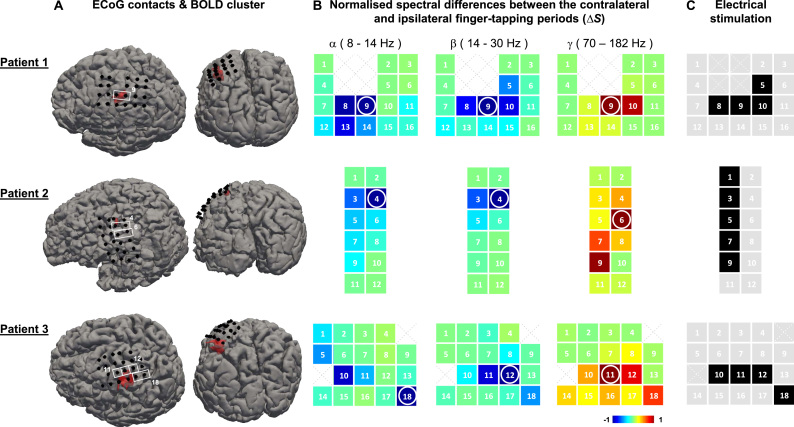
Contact pairs of interest. **A** ECoG contacts (black dots), and finger tapping BOLD increases (red). **B** Spectral differences for contralateral and ipsilateral (to the icEEG contacts) finger tapping periods. COI_α_, COI_β_ and COI_γ_ are highlighted with white circles (left, centre and right column, respectively). **C** Pre-surgical electrical stimulation results. Contacts showing peaks of apparent artefactual origins (harmonic high-amplitude peaks (prominent residual gradient artefacts), or a 50 Hz (electrical component) high-amplitude peak) were not analysed, and are displayed as a dotted cross. (For interpretation of the references to color in this figure legend, the reader is referred to the web version of this article.)

At least one cluster of statistically significant finger tapping-related BOLD changes was found in the primary motor cortex of 3/7 patients (Fig. 3), allowing us to define an appropriate ROI for the following investigations. In these 3 patients, the task consisted of interleaved right- and left- finger tapping, without rest. The data from the other 4 patients were not further analysed. The icEEG implantations are illustrated in Table 1, where the black squares show the ECoG contact pairs over the motor cortex used for the analysis (bipolar montage). The relative location of the significant cluster and ECoG contacts analysed is illustrated in Fig. 4A.

### 3.2 Contacts, frequency bands, and features of interest for BOLD modelling

#### 3.2.1 Contacts of interest (COI)

A schematic illustration of the ECoG contacts analysed is shown in Table 1. The contacts pairs highlighted with black squares in Table 1 correspond to those represented as coloured squares in Fig. 4B (each square represents an ECoG time course). The COI identified for each patient and frequency of interest are highlighted with white rectangles in Fig. 4A, and white circles in the scheme shown in Fig. 4B. The magnitude of the difference between the area under the spectrum for the contralateral (to the ECoG contacts) and ipsilateral finger-tapping periods varied considerably across the motor cortex of each patient for all frequency bands of interest, as shown by the colour scale in Fig. 4B. As expected, the sign of these differences was positive for γ, and negative for α and β.

#### 3.2.2 Patient-specific frequency bands

**Fig. 5:**
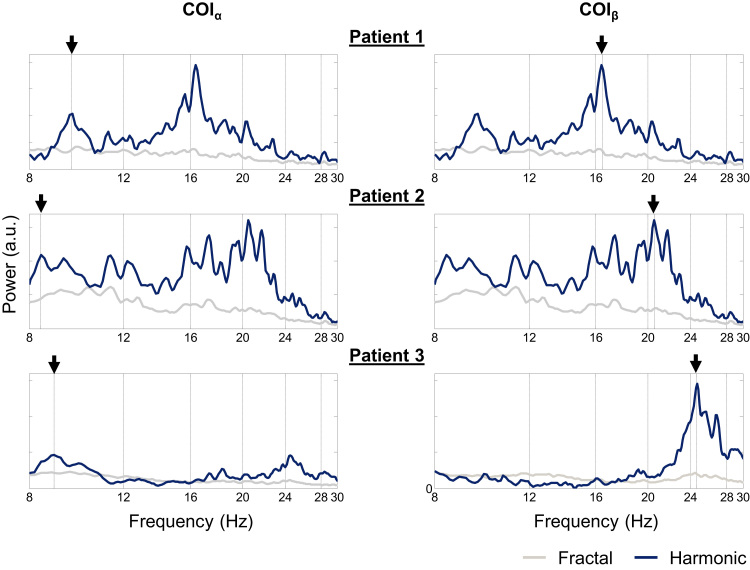
Patient-specific α and β bands of interest. Fractal and harmonic spectra for the ipsilateral finger tapping periods, computed using a coarse-graining spectral analysis (CGSA) (Yamamoto and Hughson, 1991), as described in He et al. (2010).

The fractal and harmonic power spectra, obtained for the COI_α_ and COI_β_ of each patient, and the respective α, β, and γ power peaks, marked with a black arrow, are shown in Fig. 5.

#### 3.2.3 ECoG-derived BOLD predictors

**Fig. 6:**
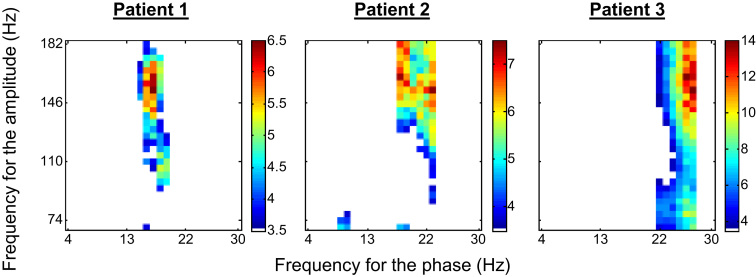
{ α, γ } and { β, γ } frequency pairs of interest. Phase–amplitude comodulogram plots (z-scored PAC strength values) for the patient-specific α and β bands (*p* < 0.05; corrected for multiple comparisons using Bonferroni criterion).

The z-scored PAC strength values for the frequency-pairs that showed a significant PAC effect (*p*<0.05; Bonferroni correction) are shown in Fig. 6. No significant PAC effect was found for the α band of patient 1 and 3. Therefore, no PAC_αγ_ regressors were defined for these patients and only their PAC_βγ_ regressors were considered in the subsequent analyses.

### 3.3 BOLD model definition

**Fig. 7:**
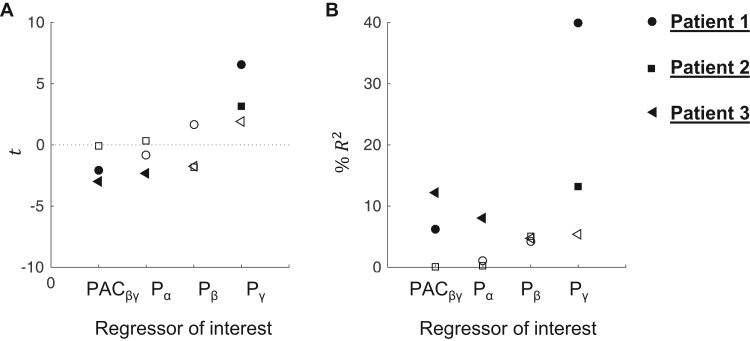
BOLD signal changes GLM results. **A***t*-value (t) for each regressor of interest (PAC_βγ_, P_α_, P_β_, and P_γ_). Filled shapes represent a *t*-value with *p*<0.05. **B** Percentage of variance explained, ${\%R}^{2}={100\times t}^{2}/(t^{2}+\mathrm{DF})$, by each regressor of interest. DF is number of degrees of freedom for the *t*-statistic. Different shapes represent different patients.

The t-values obtained for the PAC_βγ_, P_α_, P_β_, and P_γ_ effects, after estimating the models MPAC_βγ_, MP_α,βγ_, MP_β,βγ_, MP_γ,βγ_, respectively, are shown in Fig. 7. All models comprise exactly the same number of regressors – see Fig. 2. Since each effect of interest was orthogonalised with respect to all others, the t-value of each effect represents the statistical significance of the amount of variance of the BOLD signal amplitude that is explained by the effect of interest (indicated in the x-axis) in addition to a linear combination of the others (all but that of interest). Consequently, the four columns (one per effect of interest) in Fig. 7 express the following questions: Column PAC_βγ_: “Is it worth including PAC_βγ_ in a model that has P_α_, P_β_, and P_γ_?”; Column P_α_: “Is it worth including P_α_ in a model that has PAC_βγ_, P_β_, and P_γ_?”; Column P_β_: “Is it worth including P_β_ in a model that has PAC_βγ_, P_α_, and P_γ_?”; Column P_γ_: “Is it worth including P_γ_ in a model that has PAC_βγ_, P_α_, and P_β_?”.

The following regressors explained a significant amount of additional variance of the amplitude of the BOLD signal: PAC_βγ_ in 2/3 patients, and P_α_ in 1/3 patients, and P_γ_ in 2/3 patients. In particular, while in the case of patient 3, the results suggest that it is worth to include P_α_ in a model that has the strength of PAC_βγ_ and the powers of β and γ bands, in the cases of patient 1 and 2, the results suggest that it is not.

Regarding patient 2’s four α phase – γ amplitude coupling models (MPAC_αγ_, MP_α,αγ_ , MP_β,αγ_, MP_γ,αγ_), P_γ_ was the only effect that explained additional variance (results not shown).

We confirmed that PAC_αγ_, PAC_βγ_, P_α_, and P_β_ were negatively, and P_γ_ was positively, correlated with the amplitude of the BOLD signal, in 3/3 patients (Fig. S1).

Let us denote the linear Pearson correlation coefficient between A and B as corr(A,B). Fig. S1 shows that the absolute value of $\mathrm{corr}\left(P_{\alpha },\mathrm{BOLD}\right)\left(P1:-0.69\mathrm{;P}2:-0.56\right)$ is larger than that of $\mathrm{corr}({\mathrm{PAC}}_{\beta \gamma },\mathrm{BOLD})(P1:-0.32\mathrm{;P}2:-0.53)$ for patients 1 and 2. However, the absolute values of $\mathrm{corr}\left(P_{\alpha },P_{\beta }\right)\left(P1:0.63\mathrm{;P}2:0.66\right)$ and $\mathrm{corr}\left(P_{\alpha },P_{\gamma }\right)\left(P1:-0.70\mathrm{;P}2-0.59\right)$ are also larger than those of $\mathrm{corr}(P_{\alpha },{\mathrm{PAC}}_{\beta \gamma })(P1:0.40\mathrm{;P}2:0.53)$ for the same patients. Therefore, P_α_ is closer to P_β_ and P_γ_ than it is to PAC_βγ_. While PAC_βγ_ was found to explain variance of the amplitude of the BOLD signal in addition to a linear combination of P_α_, P_β_, and P_γ_, in 2/3 cases; P_α_ was found to do it in addition to a linear combination of PAC_βγ_, P_β_, and P_γ_, in 1/3 cases (Fig. 7). This suggests that the variance of the amplitude of the BOLD signal explained by P_α_ can be equally well explained by a linear combination of P_β_, P_γ_ and PAC_βγ;_ while that explained by PAC_βγ_ cannot be so well explained by a linear combination of P_α_, P_β_, and P_γ_.

## 4 Discussion

This is the first study focused on the relationship between the EEG and BOLD signals using invasive EEG and fMRI data simultaneously acquired in humans; previous studies have used either LFP and fMRI data simultaneously recorded in animals, or ECoG and fMRI data sequentially recorded in humans. In line with these previous studies, we found positive correlation coefficients for the HF (>70 Hz) EEG activities, and negative correlation coefficients for the LF (4–30 Hz) EEG activities.

This is also the first study correlating ongoing fluctuations in the PAC strength with ongoing fluctuations in the amplitude of the BOLD signal. In line with previous studies reporting that PAC is augmented during rest preceding and/or following movement, and decreased during movement execution (Miller et al., 2012, Yanagisawa et al., 2012), we found that both PAC_αγ_ and PAC_βγ_ strengths were negatively correlated with the amplitude of the contralateral finger-tapping related BOLD changes simultaneously recorded.

Finally, and, maybe, more importantly, this is the first study investigating whether the currently most commonly used EEG power-based model of the BOLD signal can be improved by adding the ongoing fluctuations on the strength of PAC_αγ_ or PAC_βγ_. For this, we tested whether single-trial estimates of the strength of PAC explained variance in addition to single-trial power fluctuations in the three EEG frequency-bands that shown finger–tapping related power changes (α, β, and γ). This investigation was based on previous studies using either LFP and fMRI data simultaneously recorded in animals, or ECoG and fMRI data sequentially recorded in humans, which have consistently found that the amplitude of the BOLD signal is better predicted by the power of the EEG signal in multiple frequency bands when compared with the γ band alone (Conner et al., 2011, Magri et al., 2012, Scheeringa et al., 2011). We found that the strength of PAC_βγ_ explained variance of the amplitude of the BOLD signal that was not explained by a combination of α, β, and γ band powers.

The closest to our study is that by Miller et al. (2012), who used ECoG and BOLD fMRI data sequentially recorded in two patients performing the same finger-tapping task. They investigated the spatial overlap between the statistically significant finger-tapping related BOLD changes (found with a whole-brain GLM analysis, using the task design boxcar regressor) and the finger-tapping related ECoG power and PAC strength changes; the finger-tapping related ECoG power and PAC strength changes were computed as the ratio between the power / PAC strength during movement and at rest, and the absolute PAC strength at rest, for every contact over the motor cortex, each resulting in a unique value per contact. Therefore, Miller et al. (2012) did not study the ongoing relationship between the strength of PAC and the amplitude of the BOLD signal, as we have done here.

### 4.1 ECoG PAC strength, power and the BOLD signal amplitude

In line with previous studies reporting that PAC is augmented during rest preceding and/or following movement, and decreased during movement execution (Miller et al., 2012, Yanagisawa et al., 2012), we found that both PAC_αγ_ and PAC_βγ_ strengths were negatively correlated with the amplitude of the finger tapping related BOLD changes. Yanagisawa et al. (2012) used ECoG to investigate movement-related PAC in the human sensorimotor cortex, and found that the α (10–14 Hz) phase was strongly coupled to the high-γ (80–150 Hz) amplitude in the waiting period (>2 s before execution), at the contacts with movement-selective high-γ amplitude during movement execution; but attenuated at the time of movement execution, suggesting that γ was “released” from the phase of α, to build a motor representation with phase-independent activity. Similarly, Miller et al. (2012) found a strong coupling between the β phase and the γ broadband spectral changes (Manning et al., 2009, Miller, 2010), especially in peri-central motor areas; this coupling was present during rest, but selectively diminished during movement, along with the amplitude of the β activity.

In line with previous studies using ECoG and fMRI data sequentially acquired in humans during sensory-motor (Hermes et al., 2012, Siero et al., 2013) or cognitive (Conner et al., 2011, Khursheed et al., 2011, Kunii et al., 2013) functions, we found that the power of the two LF bands, α and β, was negatively correlated with the amplitude of the contralateral (to the icEEG contacts) finger-tapping related BOLD changes, while the power of the γ band was positively correlated with them (Fig. S1).

#### 4.1.1 BOLD signal variance explained by ECoG PAC and power

We found that the PAC_βγ_ strength explained a significant amount of variance of the amplitude of the finger tapping related BOLD changes in addition to α, β, and γ band powers, in general. The PAC strength has been found to be entrained to behavioural events, dynamically and independently modulated in multiple task-relevant areas (Tort et al., 2008), and strongly correlated to the level of performance in a learning task (Tort et al., 2009). The PAC phenomenon combines information regarding both LF and HF electrophysiological activities, and it has been hypothesised to be an efficient mechanism to integrate fast, spike-based computation and communication with slower, external and internal state events, guiding perception, cognition, and action (Canolty and Knight, 2010). The phase of the LF activities (δ, θ, α, or β), in turn, has been shown to play an important role in the amplification of sensory inputs (Fries et al., 2002, Lakatos et al., 2005, Lakatos et al., 2007, Womelsdorf et al., 2006), attention (Fries, 2001, Lakatos et al., 2008), and behavioural responses (Jones et al., 2002, Praamstra et al., 2006, Womelsdorf et al., 2006, Lakatos et al., 2008, Schroeder and Lakatos, 2009), and it has been hypothesised to modulate cortical excitability (Fries, 2005, Lakatos et al., 2013, Lisman and Jensen, 2013, Pachitariu et al., 2015, Reig et al., 2015). At a small-scale level, the neuronal response to a particular stimulus seems to be dependent on its timing relative to the phase of the ongoing LF activity (Lakatos et al., 2013). At a large-scale level, the effective gain of long-range communication across brain areas seems to be modulated by the phase of the ongoing LF activity (Voytek et al., 2015). Therefore, PAC strength and LF band power fluctuations may have different neurophysiological origins (e.g.: they may result from the activity of different populations of neurons or from different behaviours of the same population). Different origins might be associated with different metabolic demands, which would explain the independent variance of the amplitude of the BOLD signal explained by the PAC_βγ_ strength and β band power. Interestingly, if the β activity is, in some way, equivalent to the θ activity, our hypothesis is compatible with “the θ–γ neural code” theory (Lisman and Jensen, 2013), according to which different ensembles of cells are active at different γ cycles within the θ cycle.

While, in general, PAC_βγ_ explained a significant amount of BOLD signal variance in addition to a combination of α, β, and γ band powers; the β band power did not in addition to a combination of PAC_βγ_ strength, α, and γ band powers. Magri et al. (2012) used LFP and BOLD data simultaneously recorded in the visual cortices of anesthetised monkeys during spontaneous activity to investigate the statistical dependency between the two signals; these authors found that while the γ (40–100 Hz) power was the most informative about the amplitude of the BOLD signal, both α and β band powers carried additional information, largely complementary to that carried by the γ band power. Since Magri et al. (2012) did not take into account the strength of PAC_βγ_, we can hypothesise that the variance explained by the β band power in addition to the γ band power, found by Magri et al. (2012), is better explained by the strength of PAC_βγ_, in our data.

We performed an additional analysis, similar to that described here, using data recorded during resting-state sessions (instead of finger-tapping task sessions). The PAC strength regressors computed for this additional analysis did not explain significantly variance of the amplitude of the resting-state BOLD changes in addition to a combination of the α, β, and γ band powers. This is probably explained by the fact that the strength of PAC during these resting-state sessions was weaker than that during the task sessions.

To conclude, our findings suggest that including both PAC strength and power based regressors, or even the PAC strength regressor instead of the respective LF power regressor, is likely to increase the sensitivity of the BOLD signal model, in circumstances where strong PAC strength fluctuations are observed.

### 4.2 Methodological aspects

#### 4.2.1 Electrical stimulation results and contacts of interest (COI)

During the pre-surgical evaluation, the clinicians performed an electrical stimulation study to map the motor function of each patient, i.e., to determine which ECoG contacts covered functional motor areas; the results of this study are shown in Fig. 4C. By comparing Fig. 4B and Fig. 4C, we confirm that the largest differences between the areas under the contralateral and ipsilateral spectra (${∆S}_{\alpha }$, ${∆S}_{\beta }$, and ${∆S}_{\gamma }$) were found at, or nearby, the motor function related ECoG contact pairs identified by electrical stimulation. The good spatial concordance between the electrical stimulation findings and the largest ${∆S}_{\alpha }$, ${∆S}_{\beta }$, and ${∆S}_{\gamma }$ corroborate our initial assumption that these differences are a good criterion to select finger tapping related ECoG time courses.

#### 4.2.2 Patient-specific frequency bands

Only a natural concentration of power around a particular frequency (“a peak”; an indication of rhythmic activity (Lopes da Silva, 2013)) in the time-frequency decomposition of the electrophysiological signal enables a meaningful interpretation of the phase and therefore of the PAC phenomenon (Aru et al., 2014). We use the harmonic component of the EEG power spectra to find the peaks of the task-related and patient-specific α and β activities to guarantee that we were investigating the phase of truly rhythmic activities. The γ band of interest was kept as the interval [70–182] Hz because no obvious peak was found in this frequency band for the COI_γ_ harmonic power spectrum. Interestingly, recent studies showed that the icEEG asynchronous (broadband) activity in the γ band is a better predictor of the BOLD signal in comparison to the synchronous activity in the same frequency band (Winawer et al., 2013, Nguyen et al., 2015). These findings suggest that restricting our analysis to a narrower γ band, even if it comprises mainly rhythmic activity, would reduce the significance of the correlation eventually found between the γ band power and the amplitude of the BOLD signal.

#### 4.2.3 Hemodynamic response function

We used the simplest possible model for the relationship between the amplitude of the BOLD signal and the power and PAC strength of the electrophysiological signal. Specifically, we assumed that the former is linearly proportional to the latter, and hence may be obtained through convolution with the HRF. We sought to be consistent with the previous fundamental studies on the local electrophysiological correlates of the BOLD signal (Goense and Logothetis, 2008; Magri et al., 2012; Nir et al., 2007; Scheeringa et al., 2011). A previous study found that the peak of the BOLD signal amplitude information carried by the β band power preceded that of the α and γ band powers by 0.5 s (Magri et al. 2012). However, the same group had previously reported a maximal coupling between the BOLD and EEG signals at a lag of 4–5 s (Murayama et al., 2010), which is consistent with the canonical HRF (peaking at ~5 s). The authors argued that the small difference in time lags between Magri et al. (2012) and Murayama et al. (2010) may be attributed to the longer inter-volume time used in the earlier study (2 s) when compared to that used in their later study (0.5 s). Therefore*,* the use of the canonical HRF (peaking at ~5s) seems perfectably acceptable, especially when using a longer TR as we have done here (3 s).

#### 4.2.4 Epoch duration for PAC computation

As a supplementary analysis, we investigated the influence of the duration of the PAC estimation epoch (to this point, 15 s) in our findings (results shown in Fig. S2). We used six different epoch durations: 3, 5, 7, 9, 11, 13 s; starting with 3 s because that is the temporal resolution of the BOLD signal. The results of this supplementary analysis (Fig. S2) show the trend previously seen in Fig. 7, i.e., for the 15 s epoch duration.

#### 4.2.5 Technical limitations

In 1/7 patients, the gradient artefact corrupting the icEEG data was not possible to remove due to the saturation of the amplifier used to record these data. In the remainder patients (6/7), finger tapping related ECoG power changes in the γ band (${∆S}_{\gamma }>0$) were actually observed, suggesting that these patients performed the task to some degree. However, statistically significant finger-tapping related BOLD changes were only found in three of them. This observation suggests that the task design was not powerful enough to lead to significant BOLD changes (while patient 1, 2, and 3 performed 5 blocks of contralateral finger tapping, the remainder patients performed a maximum of 3 due to the intercalation with rest), and/or that there was an apparent absence of BOLD changes despite the presence of neuronal activity, may be due to partial volume effects in fMRI measurements, a consequence of the limited spatial resolution of the fMRI acquisition, and/or fMRI signal dropout and diminished SNR in the surroundings of the icEEG contacts, a consequence of magnetic susceptibility and shielding effects caused by the presence of these metallic contacts.

In 2/3 patients, we found a spatial displacement of 2–3 cm between the ECoG and BOLD responses (Fig. 4), which contrasts with the good spatial agreement previously reported at 7 T (Siero et al., 2013). We suspect that our finger-tapping related BOLD clusters are more spatially restricted and slightly displaced from the strongest ECoG power changes than those shown by Siero et al. (2013) due to the technical differences between the two studies. In accordance with the findings of our safety work (Carmichael et al. 2012), our approach has also been to acquire fMRI data at a low magnetic field (1.5 T) to limit the health risks (tissue overheating and potential excitation) which are caused by the exposure of the closed circuits formed by the EEG leads, amplifier and patient to the radio-frequency (RF) pulses used to excite the magnetisation of the protons in the fMRI acquisition sequence. We specified a TR of 3 s in our protocol in order to provide whole brain coverage (shorter TR values would allow only partial coverage), because the implantation scheme varies significantly from patient to patient (due to the different clinical histories and aimed investigations) and we aimed to run the same sequence on all patients. The low magnetic field (1.5 T) used in our study is in sharp contrast with that used in Siero et al. (2013) (7 T), who took advantage of a comparatively higher temporal and spatial SNR in order to achieve better spatial resolution (1.5×1.5×1.5 mm while ours is 3×3×2.5 mm) and temporal resolution (880 ms while ours is 3000 ms). Adding to these technical disadvantages, we must also consider the signal dropout and diminished SNR in the surroundings of the icEEG contacts which did not affect Siero et al. (2013). These differences in the data acquisition protocol are likely to increase their study sensitivity in comparison to ours and therefore to lead to wider and easier to detect finger-tapping BOLD changes.

The quality of the fMRI data achieved with the setup used here was extensively quantified and described in our previous study - Carmichael et al. (2012). In brief, the amplitude of the GE-EPI signal is around 70% of its whole brain average value at ~5 mm away from the icEEG contact and practically 100% at ~10 mm away from it; the % of signal loss varies considerable across contacts and depends on the electrode orientation relative to the MRI scanner axes (there are greater losses for contacts with a vector normal to the grid surface parallel to B_0_) (Carmichael et al., 2012).

Even though the quality of icEEG and BOLD data sequentially acquired may be better than that of data simultaneously acquired, this study together with Murta el al. 2016 show that we can further investigate the relationship between these two signals using data simultaneously acquired in humans. More importantly, simultaneous multimodal acquisitions are the only way of guaranteeing that the data relate to the same (neuronal) phenomenon. Thereby, these acquisitions provide a theoretical sensitivity benefit. In general, inter-event and inter-session variability due to different performances, habituation effects, plasticity, or uncontrolled variations in the response to the stimulation paradigm cannot be investigated using sequentially acquired data (Villringer et al., 2010). In particular, the cooperation of patients (i.e. their will to perform the finger tapping exactly when asked to do it) will not affect dramatically studies using multimodal data simultaneously acquired but it will affect studies using sequentially acquired data, potentially resulting in a lower correlation between the two signals that is not explained by their decoupling. The access to ECoG and fMRI data simultaneously acquired was a good opportunity to further investigate the relationship between the two signals and complement previous studies that have used data sequentially acquired.

The potential safety risks associated with icEEG and fMRI data simultaneous recordings were extensively evaluated and discussed in Carmichael et al. (2010). Theoretically, the main risks of recording icEEG data simultaneously with fMRI data are the mechanical forces on the icEEG electrodes caused by transient magnetic effects, the heating of tissues due to interaction with the pulsed RF fields, and the stimulation of tissues due to interactions with the switched magnetic gradient fields. In practice, the greatest effective risk was found to be the RF-induced tissue heating in the proximity of the depth and grid electrode contacts. This heating was limited by using a head coil, adding connecting cables, carefully controlling their length and position, and using a low-SAR sequence (Carmichael et al., 2010).

#### 4.2.6 Potential improvements

Our experiments have been performed after the invasive pre-surgical investigation, which uses clinically certified (by the relevant medical device regulatory bodies) ECoG and depth icEEG electrodes, designed to have optimal EEG recording and surgical proprieties for medical purposes and not to have optimal MR imaging properties. Using icEEG contacts with better magnetic proprieties (i.e. magnetic susceptibilities closer to the brain tissue, for instance) would probably improve the quality of the fMRI data and therefore the sensitivity of these kind of studies; however, this technical modification would be a significant undertaking.

A higher temporal sampling rate would increase the number of fMRI data points per condition (contralateral finger tapping, ipsilateral finger tapping, rest), which is likely to facilitate the detection of finger tapping related BOLD changes. It would also allow us to exploit better the temporal richness of the EEG signal; however, only to the extent allowed by the inherently slow dynamics of the BOLD signal. Better fMRI temporal and spatial resolutions can be achieved with higher magnetic fields that however bring safety concerns that need to be carefully studied and minimised. Improving the quality of the EEG data simultaneously recorded with fMRI, by minimising the temporal variability of any residual MR-related artefacts, while maximising the cut-off frequency of the hardware low-pass filter used, would allow us to explore higher EEG frequency ranges and potentially improve the accuracy of the EEG-derived features.

## 5 Conclusion

Using ECoG and fMRI simultaneously recorded in humans, we found that the amplitude of the BOLD signal was negatively correlated with both PAC strength and power of the lower α and β EEG frequencies, and positively correlated with the power of the higher γ EEG frequencies. These findings were consistent with previous studies using LFP and fMRI simultaneous recorded in animals, and ECoG and fMRI data sequentially recorded in humans. More importantly, we found that the PAC strength explained variance of the amplitude of the BOLD signal in addition to α, β, and γ band powers, which not only suggests that we may increase the sensitivity of EEG-informed fMRI studies by taking the PAC strength into account, but also that the power of LF activities and the strength of PAC may have different neurophysiological origins, and may therefore have different functional roles worth to keep investigating.

## Figures and Tables

**Table 1:** EcoG implantation characterisation: types of electrodes, number of contacts per electrode, and implantation scheme. The ECoG contact pairs analysed (over the motor cortex) are highlighted with numbered black squares. FLE: Frontal lobe epilepsy, R: right, L: left, A: anterior, M: medial, P: posterior, I: inferior, and S: superior.

		**Implantation scheme**
**Patient ID**	1	
**Type of epilepsy**	FLE	
**Anatomical location of electrodes**	- L pre/postcentral gyrus - L supramarginal gyrus - I (IFG) and M (MFG) frontal gyri	
**Type of electrodes**	two 6-contact strips, one 8x8 contact grid, one 2x8 contact grid	
**Patient ID**	2	
**Type of epilepsy**	FLE	
**Anatomical location of electrodes**	- L frontal lobe (laterally and inferiorly) - L M (MFG) and I (IFG) frontal gyri - L temporal lobe	
**Type of electrodes**	one 6x8 contact grid, two 2x8 contact grids, one 4x8 high-density contact grid, two 6-contact strips, two 6-contact depths	
**Patient ID**	3	
**Type of epilepsy**	FLE	
**Anatomical location of electrodes**	- L frontal and parietal convexity - L frontal pole - L S frontal gyrus (SFG) - L I frontal gyrus - L mesial frontal surface	
**Type of electrodes**	one 8x8 contact grid, one 2x8 contact grid, one 8-contact strip, one 6-contact strip, one high-density 4x8 contact grid	

## Appendix A Supplementary material

Supplementary material **Fig. S1** Cross-correlation matrices. Pairwise Pearson's linear correlation coefficients for all ECoG-derived regressors and BOLD time course of interest.

.

Supplementary material **Fig. S2** BOLD signal changes GLM results. t-values for the PAC_βγ_, P_α_, P_β_, and P_γ_ regressors. Different colours represent different data epoch durations for the computation of the continuous PAC regressor. Different shapes represent different patients. Filled shapes represent t-values with *p*<0.05.
