# Supplementary figures and images for: Phase–amplitude coupling and the BOLD signal: A simultaneous intracranial EEG (icEEG) - fMRI study in humans performing a finger-tapping task
Source: Neuroimage. 2017 Feb 1;146:438–51. doi: 10.1016/j.neuroimage.2016.08.036 (PMC5312786; doi:10.1016/j.neuroimage.2016.08.036)

Patient 1

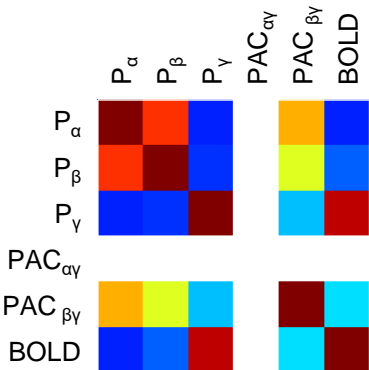

Patient 2

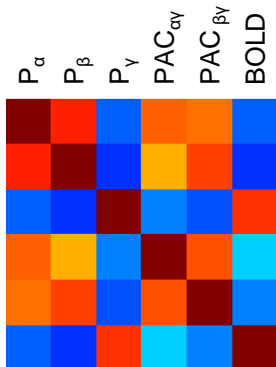

Patient 3

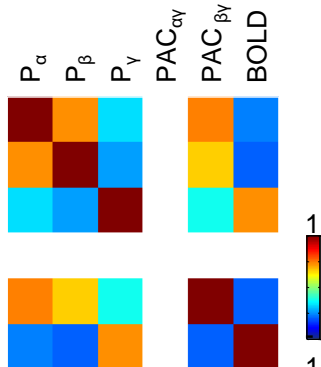

Supplement: Supplementary file 1 — Supplementary material Fig. S1 Cross-correlation matrices. Pairwise Pearson's linear correlation coefficients for all ECoG-derived regressors and BOLD time course of interest. [file mmc1.pdf]

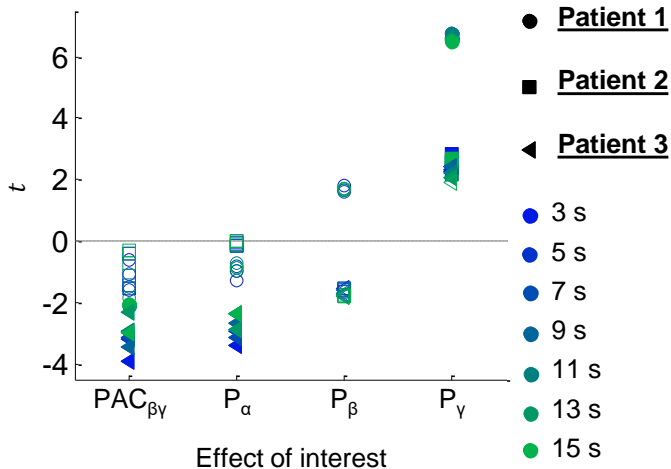

Supplement: Supplementary file 2 — Supplementary material Fig. S2 BOLD signal changes GLM results. t-values for the PACβγ, Pα, Pβ, and Pγ regressors. Different colours represent different data epoch durations for the computation of the continuous PAC regressor. Different shapes represent different patients. Filled shapes represent t-values with p<0.05. [file mmc2.pdf]
